# Screening for psychological distress in childhood and mental health-related hospital attendance among young adults

**DOI:** 10.23889/ijpds.v9i5.2513

**Published:** 2024-09-18

**Authors:** Gergo Baranyi, Katie Harron, Nasir Rajah, Emla Fitzsimons

**Affiliations:** 1 Centre for Longitudinal Studies, UCL Institute of Education, University College London; 2 Population, Policy & Practice Department, UCL GOS Institute of Child Health, University College London

## Objectives

The prevalence of mental disorders is high and increasing among children and young people. Investigating the relationship between self-reported poor mental health and mental health service utilisation can provide evidence on the benefits of universal screening, but longitudinal data is rare.

## Approach

Data from a nationally representative English cohort (Next Steps) were linked to NHS Hospital Episode Statistics. GHQ-12 assessed psychological distress in Next Steps at age 15; participants were followed until their first mental health-related hospital presentations or were censored at the end of the study (age 27). Cox proportionate hazard models with survey weights estimated associations.

## Results

Out of 4058 young people included in the analyses, 19% reported high levels of distress at age 15. During the 12-year follow-up, 5.3%, 2.9% and 2.7% of the participants had at least one mental disorder, drug/alcohol misuse and self-harm presentation, respectively, and 4.2% had a mental health treatment. Higher GHQ-12 scores were associated with mental disorder presentations (HR=1.10, 95% CI:1.04-1.16), and mental health treatments (HR=1.14, 95% CI:1.08-1.20). Associations were weaker for young people living in deprived areas, or if their main parent had lower education.

## Conclusions

Adolescent psychological distress predicts subsequent hospital attendance in young adulthood, but there are treatment gaps in service utilisation among more disadvantaged individuals. Detecting youth with mental health difficulties through universal screening, may facilitate early intervention, improve life-course outcomes, and reduce secondary healthcare use.

## Implications

TLinked cohort and administrative data can improve our understanding on long-term healthcare utilisation and treatment needs.

